# Fungal diversity and mycotoxins in retail polished and unpolished rice in Thailand

**DOI:** 10.3389/fnut.2026.1828938

**Published:** 2026-06-25

**Authors:** Supeecha Waiya, Saranya Poapolathep, Donnaya Thanakitpipattana, Nattawut Boonyuen, Onuma Piasai, Johanna F. Gremmels, Sarah De Saeger, Antonio F. Logrieco, Amnart Poapolathep

**Affiliations:** 1 Department of Pharmacology, Faculty of Veterinary Medicine, Kasetsart University, Bangkok, Thailand; 2 National Center for Genetic Engineering and Biotechnology (BIOTEC), National Science and Technology Development Agency (NSTDA), Pathum Thani, Thailand; 3 Department of Plant Pathology, Faculty of Agriculture, Kasetsart University, Bangkok, Thailand; 4 Institute for Risk Assessment Sciences, Faculty of Veterinary Medicine, Utrecht University, Utrecht, Netherlands; 5 Centre of Excellence in Mycotoxicology and Public Health, Faculty of Pharmaceutical Sciences, Ghent University, Ghent, Belgium; 6 Xianghu Laboratory, Zhejiang Provincial Laboratory of Agriculture, Hangzhou, China; 7 Research National Council, Institute of Sciences of Food Production, Bari, Italy

**Keywords:** *Aspergillus*, LC–MS/MS, multi-locus phylogeny, mycotoxins, *Neocosmospora* (*Fusarium solani* species complex), rice, *Talaromyces*

## Abstract

Rice is a major dietary staple food across Asia, including Thailand, and is susceptible to fungal invasion and mycotoxin contamination. Because rice is consumed daily, even low-level contamination may have public-health implications. This study aimed to identify fungal diversity on unpolished and polished rice samples and to analyse the occurrence of mycotoxins in retail rice from Thailand. To this end, 50 retail rice samples (25 polished and 25 unpolished) were collected from markets across various districts of Bangkok, Thailand. Using the blotter method, 12.6% of rice proved to be contaminated by fungi with a slightly higher contamination rate observed in unpolished rice than in polished rice (13.28% and 11.92%, respectively). Morphological and molecular examination using multi-locus sequencing revealed various taxa belonging to *Aspergillus*, *Neocosmospor*a (*Fusarium solani* species complex) and *Penicillium/Talaromyces*. Chemical analysis by liquid chromatography–tandem mass spectrometry detected beauvericin (BEA; *n* = 2) and zearalenone (ZEN; *n* = 2) only in unpolished rice, with concentrations up to 1.77 μg/kg and 65.57 μg/kg, respectively. None of the samples exceeded the acceptable ZEN threshold set by the Thai Food and Drug Administration (100 μg/kg). In conclusion, the results of this study confirmed that unpolished is prone to fungal contamination originating from environmental, as well as handling during harvesting, sorting and packaging. The occurrence of mycotoxin was limited to a small percentage of unpolished rice samples. No regulated carcinogenic mycotoxins such as AFs, OTA, or FB_1_ were found. Using validated LC–MS/MS methods, only ZEN and BEA were detected at low levels in unpolished rice, and no mycotoxins above the LOQ were found in polished rice. The results show low contamination in the rice samples from Bangkok retail markets. However, further studies are needed to confirm these findings at a broader level. The multi-toxin analytical method described enables routine, validated monitoring of rice from various harvests, helping to support food safety objectives.

## 1 Introduction

Rice (*Oryza sativa* L.) is one of the world’s most important crops and a staple food for over 3 billion people, with Asia as the primary center of production and consumption (1, 2). Major producers and consumers include China, India, Bangladesh, Indonesia, Vietnam, and Thailand, collectively underpinning regional food security and global rice trade. Beyond direct consumption, rice is utilized in beverage production and in value-added products, such as rice bran and rice bran oil, which are key components of the agri-food sector (3, 4). Global rice production has increased steadily, reaching 540.93 million tons in 2024–2025, with India as the largest producer and Thailand ranking sixth worldwide (5). Thailand’s high rice genetic diversity supports adaptability and crop resilience, although recent shifts in cultivation practices and climatic changes may increase fungal contamination and associated quality and safety risks (6).

In tropical and subtropical regions, rice grains are vulnerable to fungal colonization during cultivation, drying, and storage, most commonly involving *Aspergillus*, *Penicillium*, *Talaromyces*, and *Fusarium* (*sensu lato*). Elevated moisture and temperatures, together with inadequate drying and suboptimal storage practices, promote fungal proliferation and can increase the likelihood of mycotoxin contamination in rice and rice-based products (7). Among these fungi, *Aspergillus flavus* and *A. parasiticus* are associated frequently with hot and humid environments and are well-recognized producers of aflatoxins (8). *Penicillium* species, including *P. verrucosum* and *P. citrinum*, are important post-harvest contaminants and may dominate during storage when grain moisture remains high (often >15%) (9, 10). *Talaromyces*—formerly classified within *Penicillium* and later redefined based on molecular and phylogenetic evidence (11)—includes species reported frequently from cereals (including rice) and animal feed, notably *T. funiculosus, T. pinophilus*, and *T. purpureogenus*. These species occur more frequently in humid environments, where although typically recovered at lower frequencies than other dominant genera, their incidence has been reported to increase in tropical regions (12). In addition, *Fusarium* (*sensu lato*) species are recognized widely as important contaminants of rice during both the pre-harvest and post-harvest stages (13). Field-associated species, such as *F. graminearum* and *F. fujikuroi*, commonly infect rice plants under humid conditions, contributing to diseases including bakanae and *Fusarium* head blight. In contrast, members of the *Fusarium fujikuroi* species complex (FFSC), including *F. proliferatum* and *F. verticillioides*, may persist post-harvest and proliferate during storage when moisture is not controlled adequately (14, 15). In addition, *Fusarium solani* species complex (FSSC) has been the subject to, which a taxonomic revisions have reassigned to the genus *Neocosmospora*, are increasingly isolated from cereals and plant-derived substrates (72). However, the transfer of *F. solani* and the FSSC out of the genus *Fusarium* into the relatively obscure genus *Neocosmospora* remains controversial. Geiser et al. (16) argued that this move may be scientifically unnecessary and impractical and questioned the phylogenetic arguments presented to support it. In the present study, we adopt the name *Neocosmospora* to maintain consistency with the phylogenetic framework used in our analyses.

Mycotoxins are a chemically diverse group of fungal secondary metabolites that can contaminate crops and foods. Many mycotoxins are relatively stable during processing and may persist in food matrices even after common thermal treatments. Major mycotoxins of public health concern include aflatoxins, ochratoxin A (OTA), fumonisins (FBs), trichothecenes, and zearalenone (ZEN), all of which have well-documented toxicological effects. For example, aflatoxin B_1_ (AFB_1_) is classified as carcinogenic to humans (Group 1) and is strongly associated with hepatotoxicity and hepatocellular carcinoma (17). OTA is nephrotoxic and has been linked to chronic kidney disease in certain populations (18). FBs, particularly fumonisin B_1_ (FB_1_), can affect the neural and cardiovascular system and have been associated with esophageal cancer and neural tube defects (19–21). Trichothecenes, such as deoxynivalenol (DON), induce vomiting, immunosuppression, and inhibition of protein synthesis (15, 22). ZEN has estrogenic activity and disrupts reproductive function in various animal species (9, 23, 24). In tropical regions such as Thailand, warm temperatures and high humidity promote fungal growth and mycotoxin formation, underscoring the importance of effective drying and moisture and temperature control to reduce contamination rice and cereals.

Given these concerns, the present study aimed to identify fungal diversity in unpolished and polished rice samples and to analyze the occurrence of mycotoxins in retail rice samples in Thailand. The findings are expected to provide updated evidence to support risk assessment and inform risk-based monitoring and food safety management strategies across Thailand’s rice production and distribution systems.

## 2 Materials and methods

### 2.1 Sampling

In total, 50 retail rice samples were purchased and categorized as polished (*n* = 25) and unpolished (*n* = 25). Samples were obtained without preselection from five retail department stores in Bangkok, Thailand and were sold as prepackaged products (500–1,000 g per package). The samples included different brand, rice types, producers, and distributors commonly available in retail department. Purchases were made between late 2023 and early 2024. Following purchase, all samples were transported to the laboratory and stored at 4 °C until analysis.

### 2.2 Chemicals and reagents

All chemicals and solvents were of high-performance liquid chromatography (HPLC) grade. Distilled and Milli-Q water (Millipore, Bedford, MA, USA) were used throughout the experiments. For fungal isolation and morphological examination, Potato Dextrose Agar (PDA; Difco, Detroit, MI, USA) and Carnation Leaf Agar (CLA) were used. For molecular analyses, OmniPCR Supermix (OnePCR Ultra) with fluorescent dye and DNA ladder were obtained from Bio-Helix Co., Ltd. (New Taipei City, Taiwan). Cetyltrimethylammonium bromide (CTAB), chloroform, isoamyl alcohol, isopropanol, 70% ethanol, TE buffer, PCR primers, and agarose gel were of analytical grade. For mycotoxin analysis, acetonitrile, methanol, acetic acid, and formic acid were purchased from RCI Labscan (Bangkok, Thailand). Ammonium formate, sodium citrate tribasic dihydrate, and sodium citrate dibasic sesquihydrate were obtained from Sigma-Aldrich (Taufkirchen, Germany). Magnesium sulfate (MgSO₄) was obtained from PanReac Química (Barcelona, Spain). Sodium chloride (NaCl) was obtained from KemAus (New South Wales, Australia). Octadecyl-bonded silica (C18) was purchased from Macherey-Nagel (Duren, Germany), and primary–secondary amine (PSA) from Biocomma Biotech Co., Ltd. (Shenzhen, China).

### 2.3 Detection and isolation of seed-borne Fungi

Seed-borne fungi were detected using the blotter method (25), in accordance with the International Rules for Seed Testing (26). For each rice sample, 400 grains were examined (16 Petri dishes × 25 grains per dish). Each Petri dish was lined with three layers of blotter paper moistened with sterile water to maintain adequate humidity, and the grains were evenly spaced on the surface to avoid contact between them. Plates were incubated at 25 °C under alternating 12 h light/12 h dark conditions for 7 days. After incubation, each grain was individually examined for visible fungal growth and the data obtained were used to calculate the percentage of fungal contamination for each rice sample using equation (27).


$$\begin{array}{l} \text{Frequency of occurrence}\;(\%)\\ =\frac{\mathrm{No}.\text{of seeds}\;\mathrm{on}\;\text{which}\;a\;\text{fungal species occurs}}{\text{Total number of seeds}}\times 100 \end{array}$$

### 2.4 Morphological identification of fungi

Morphological identification was carried out using culture-based methods on diagnostic media. Fungal growth observed in the blotter assay was transferred to PDA and incubated at 25 °C for 7 days, under standard seed pathology conditions for fungal isolation and culture (28). To obtain pure cultures, single-spore isolation was performed from representative colonies, and the resulting isolates were sub-cultured onto fresh PDA plates. *Fusarium*-like isolates (*sensu lato*) were further sub-cultured onto carnation leaf agar (CLA) and incubated at 25 °C for 10–14 days to induce sporulation and support morphological characterization (14). Colony characteristics, including color and pigmentation, were assessed on PDA for all isolates, while CLA was used for *Fusarium*-like isolates to promote sporulation and enable observation and measurement of conidia and associated structures (14, 29, 30). Microscopic features, including macroconidia, microconidia, conidiophores, phialides, vesicles, and conidial arrangement, were mounted on microscope slides and examined under a compound microscope at 40× magnification using published identification keys (31, 32). The frequency of each fungal taxon (genus/species) was calculated as the percentage of grains on which that taxon occurred, following Butt et al. (27).

### 2.5 Molecular identification and phylogenetic analysis of fungal isolates

Genomic DNA was extracted from actively growing mycelia obtained from cultures aged 3 days, using a CTAB based protocol (33, 34). Fungal mycelia were homogenized in CTAB extraction buffer, vortexed, and incubated at 65 °C for 1 h. chloroform:isoamyl alcohol (24:1) was added and the mixture was centrifuged at 10,000 rpm for 10 min to separate the phases. Next, the aqueous phase was transferred to a new tube and the DNA was precipitated using isopropanol, followed by centrifugation at 15,000 rpm for 5 min to precipitate the DNA. The resulting pellet was washed with 75% ethanol, air-dried, and resuspended in TE buffer (34).

Molecular identification was carried out using PCR amplification and sequencing of multiple-loci. *Aspergillus* and *Penicillium/Talaromyces* were targeted for the internal transcribed spacer region (ITS), *β*-tubulin (BenA), calmodulin (CaM), and the RNA polymerase II second largest subunit (RPB2). For *Fusarium* (*sensu lato*), ITS, translation elongation factor 1-alpha (TEF-1α) and RPB2 were amplified. The following primers were used: ITS5/ITS4 for ITS (35, 36); Bt2a /Bt2b (and Bt1 where applicable) for BenA (35); CMD5/CMD6 and/or CL1/CL2a for CaM (37, 38); 5F2/7CR for RPB2 (39, 40), and EF1/EF2 for TEF-1α (30, 41).

PCR reactions were performed in 25 μL volumes containing 0.5 μL of genomic DNA, 12.5 μL of 2 × PCR master mix, 0.5 μL (10 μM) of forward and reverse primers, and nuclease-free water to make up the volume. The cycling conditions were: ITS—95 °C for 2 min; 34 cycles of 95 °C for 60 s, 59 °C for 2 min, and 72 °C for 2 min 30 s, with the final extension at 72 °C for 10 min; BenA—94 °C for 3 min; 35 cycles of 94 °C for 60 s, 57 °C for 60 s, and 72 °C for 2 min, with the final extension at 72 °C for 10 min; CaM—94 °C for 10 min; 30 cycles of 94 °C for 50 s, 55 °C for 55 s, and 72 °C for 60 s, with the final extension at 72 °C for 7 min; RPB2—94 °C for 4 min; 34 cycles of 94 °C for 30 s, 55 °C for 30 s, and 72 °C for 60 s, with the final extension at 72 °C for 10 min; and TEF-1α —95 °C for 2 min; 35 cycles of 95 °C for 50 s, 59 °C for 50 s, and 72 °C for 60 s, with the final extension at 72 °C for 7 min. Amplicons were visualized on 1.5% agarose gels in TAE buffer and compared with a DNA ladder. Purified PCR products were sequenced by Macrogen Inc. and Apical Inc. using the same primers as for amplification. Forward and reverse chromatograms were trimmed, edited, and assembled in BioEdit v7.2 (42).

For phylogenetic analyses, concatenated multi-locus datasets were constructed: ITS+BenA+CaM + RPB2 for *Aspergillus* and *Penicillium/Talaromyces*; and ITS+TEF-1α + RPB2 for *Fusarium* (*sensu lato*). Newly generated sequences were deposited in GenBank, with their accession numbers provided in Supplementary Tables S1, S2. *Metarhizium anisopliae* ARSEF 3145 and *Metarhizium pinghaense* CBS 257.90 were used as outgroup taxa for the phylogenetic analyses of *Aspergillus*, *Penicillium*, and *Talaromyces*, whereas *Geejayessia cicatricum* CBS 125552 and *Geejayessia atrofusca* NRRL 22316 were selected as outgroup taxa for the *Fusarium* dataset. The gene datasets four-gene concatenated dataset for *Aspergillus/Penicillium/Talaromyces* and three gene dataset for *Fusarium* were aligned and assembled in BioEdit (42). Sequence alignments were generated and manually refined in BioEdit (42). Maximum likelihood (ML) analyses were performed in RAxML-HPC2 on ACCESS with 1,000 bootstrap replicates (43). Bayesian inference (BI) was conducted in MrBayes v3.2.7 for 5,000,000 MCMC generations, with appropriate burn-in applied prior to summarizing posterior probabilities (44). Phylogenetic trees were visualized and edited using FigTree v1.4.4.

### 2.6 Mycotoxin extraction from rice samples

Mycotoxin extraction from rice samples was performed following the QuEChERS-based method described by Sinphithakkul et al. (45), adapted from the original QuEChERS procedure (46). Briefly, 1 g of finely ground rice was extracted with 5 mL of Milli-Q water and 5 mL of 10% acetic acid in acetonitrile, followed by vortexing for 2–3 min. Subsequently, 2 g of MgSO₄, 0.5 g of sodium citrate tribasic dihydrate, 0.25 g of sodium citrate dibasic sesquihydrate, and 0.5 g of NaCl were added. The mixture was shaken for 1–2 min and centrifuged at 1,968 × *g* for 5 min. A 2 mL aliquot of the upper layer was transferred to a clean tube containing 300 mg MgSO_4_, 50 mg C_18_, and 25 mg PSA. After vortexing for 2–3 min, the sample was centrifuged again at 1,968 × *g* for 10 min. A 1 mL portion of the supernatant was evaporated to dryness under nitrogen and reconstituted in the initial LC–MS/MS mobile phase. The final extract was passed through a 0.22 μm syringe filter and analyzed by LC–MS/MS.

### 2.7 LC–MS/MS analysis of mycotoxins

LC–MS/MS analysis was performed using a method adapted from other published studies (45, 47). Chromatographic separation was achieved on an eclipse plus C18 column using a mobile phase consisting of (A) 5 mM ammonium formate with 0.2% formic acid in water and (B) methanol. The gradient program was set as: 0–1 min, 10% B; 1–5 min, 10%–95% B; 5–10 min, 95% B; and 10–12 min, 10% B, followed by a 3 min re-equilibration between injections. The flow rate was set at 0.4 mL/min and the injection volume was 10 μL. All mobile phases were filtered through a 0.22 μm membrane and degassed using ultrasonication before use. Detection was carried out on an Agilent 6460 triple quadrupole LC–MS/MS system (Agilent Technologies; Waldbronn, Germany) equipped with an electrospray ionization source operating in positive ion and negative ion mode under multiple reaction monitoring (MRM). Source and compound-dependent parameters were optimized using authentic standards to maximize the sensitivity and selectivity for each analyte.

### 2.8 Validation of the LC–MS/MS method for multi-mycotoxin detection

Validation of the LC–MS/MS method for multi-mycotoxin (AFB_1_, AFB_2_, AFG_1_, AFG_2_, OTA, ZEN, BEA, FB_1_, FB_2_, T_2_, DAS, ALT, CIT, DON, NIV) determination was performed according to the performance criteria of Commission Decision 2002/657/EC (48). The validation parameters consisted of linearity, accuracy, precision, recovery, stability, limits of detection (LOD), limits of quantification (LOQ), and matrix effects. LOD and LOQ were calculated based on signal-to-noise ratios of 3:1 and 10:1, respectively. The LODs were in the ranges 0.15–25.0 μg/kg, and 0.20–28.2 μg/kg, whereas the LOQs were in the ranges 0.50–83.33 μg/kg, and 0.69–92.8 μg/kg, for polished rice and unpolished rice, respectively (Table 1). Linearity was evaluated using matrix-matched calibration curves prepared by fortifying blank samples across the working concentration range. Accuracy and intra-day precision were assessed using spiked samples analyzed in seven replicates at three quality control (QC) levels. Inter-day precision was determined by analyzing QC samples on five separate days. Matrix effects were evaluated by comparing calibration curve slopes obtained from post-extraction spiked samples with those prepared in pure solvent. Identification of positive samples was based on the agreement of retention times with those of reference standards, together with the detection of at least two MRM transitions per analyte and ion ratios being within the tolerance limits specified in Commission Decision 2002/657/EC.

**Table 1:** Limits of detection (LOD), limits of quantification (LOQ), and linearity (*R*^2^) of the proposed method for mycotoxin analysis in polished and unpolished rice.

Analyte	Groups of polished rice			Groups of unpolished rice		
	LOD (µg/kg)	LOQ (µg/kg)	R^2^	LOD (µg/kg)	LOQ (µg/kg)	*R* ^2^
AFB_1_	0.30	0.98	0.9965	0.35	1.17	0.9980
AFB_2_	0.21	0.71	0.9975	0.27	0.86	0.9982
AFG_1_	0.15	0.50	0.9980	0.22	0.73	0.9990
AFG_2_	0.20	0.67	0.9982	0.25	0.83	0.9955
OTA	0.18	0.60	0.9997	0.20	0.69	0.9990
T_2_	0.80	2.67	0.9935	1.55	5.17	0.9950
DAS	2.00	6.67	0.9941	2.75	9.17	0.9980
ZEA	8.25	27.50	0.9953	12.5	41.67	0.9965
FB_1_	0.31	1.04	0.9990	0.47	1.55	0.9995
FB_2_	0.44	1.48	0.9978	0.66	2.20	0.9980
BEA	0.20	0.68	0.9995	0.28	0.93	0.9990
CIT	0.65	2.17	0.9990	0.85	2.83	0.9978
ALT	0.55	1.85	0.9977	0.70	2.33	0.9985
DON	7.80	25.11	0.9935	12.5	41.67	0.9950
NIV	25.0	83.33	0.9928	28.2	92.8	0.9945

## 3 Results

### 3.1 Fungal incidence in rice grains

Using the blotter method, 2,520 contaminated grains were detected among 20,000 grains examined, corresponding to an overall contamination rate of 12.6%. Based on rice type, 1,192 contaminated grains were found in polished rice (11.92%), while 1,328 contaminated grains were found in unpolished rice (13.28%) (Table 2). This pattern may be related to the rice decortication process, in which the outer layers of the grain, which are often more contaminated, are removed. Decortication is a mechanical process that removes the bran and germ layers from the endosperm to produce white rice, typically by abrasive milling. These findings suggest that the outer grain layers may act as a reservoir of fungal propagules and indicate the importance of appropriate post-harvest handling and milling processes to reduce fungal contamination in rice intended for consumption.

**Table 2:** Incidence of fungal contamination in polished and unpolished rice samples.

Variety of rice sample	Total tested rice kernels (*n*)	Fungal incidence	
		Number of contaminated rice kernels (*n*)	Contamination (%)
Polished	10,000	1,192	11.92%
Unpolished	10,000	1,328	13.28%
Total	20,000	2,520	12.6%

Contamination (%) = (Contaminated kernels/total kernels examined) × 100.

### 3.2 Fungal Isolation from rice samples

**Figure 1:**
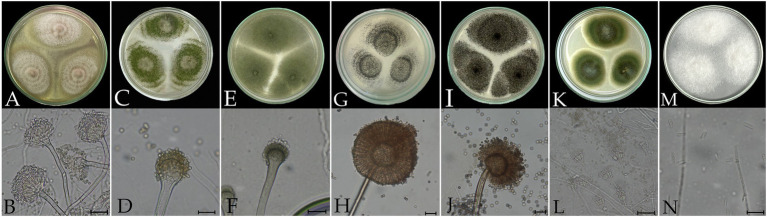
Colony morphology and microscopic characteristics of representative fungal isolates. Isolates were cultured on PDA at 25 °C for 7 days and microscopic features showing conidial structures: **(A,B)** *Aspergillus aureoterreus* RW12; **(C,D)** *A. oryzae* RW09; **(E,F)** *A. fumigatus* RC10; **(G,H)** *A. tubingensis*; **(I,J)** *A. awamori*; **(K,L)** *Talaromyces pinophilus* RW08; **(M)** *Neocosmospora pseudensiformis* RC15; **(N)** *N. pseudensiformis* RC15 cultured on carnation leaf agar. Scale bars = 20 μm.

In total, 50 commercial rice samples were obtained from department stores in Thailand to represent rice products commonly available to consumers. From the 50 rice samples (polished, *n* = 25; unpolished, *n* = 25) examined, a total of 20 fungal isolates were successfully recovered from grains showing visible fungal infection after incubation using the blotter method. Preliminary morphological identification revealed that *Aspergillus* was the most frequently detected genus, representing 18 of 20 isolates (90%), followed by *Penicillium/Talaromyces* (1 isolate; 5%) and *Fusarium* (1 isolate; 5%). In total, 11 isolates were recovered from the polished rice and 9 from the unpolished rice. The predominance of *Aspergillus* spp. in both rice types may have reflected the strong adaptation of these species to post-harvest storage conditions, particularly those of elevated grain moisture and water activity that favor colonization. This observation was consistent with other published reports that indicated *Aspergillus* species were commonly associated with rice and other stored cereals under hot and humid environments (49, 50). In this first stage of identification, phenotypic assessments were conducted based on cultural traits and microscopic morphology, with the representative features of each isolate presented in Figure 1.

### 3.3 Molecular and phylogenetic identification of fungal isolates from rice samples

Commonly, morphological characterization is used in practice for fungal identification at the genus level; however typically, species-level resolution requires molecular data (51). In the present study, representative fungal isolates were identified using multi-locus sequencing, with preliminary sequence similarity assessed based on BLAST searches against the NCBI nucleotide database, followed by phylogenetic confirmation using curated reference sequences. For *Aspergillus* and *Penicillium/Talaromyces*, four loci (ITS, BenA, CaM, and RPB2) were analyzed, whereas for *Fusarium* (*sensu lato*) isolates, ITS, TEF-1α, and RPB2 were used.

Nine representative isolates were subjected to molecular identification, with the BLAST results showing high sequence similarity to their corresponding species, as well as being congruent with the morphological observations. For example, isolate RW12 showed 100% (ITS), 99.6% (BenA), 97.6% (CaM), and 98.6% (RPB2) identity to *Aspergillus aureoterreus* (Supplementary Table S3). Isolate RW15 showed 99.7% (ITS), 99.8% (CaM), and 100% (RPB2) identity to *A. fumigatus* (Supplementary Table S3). The *Fusarium* (*sensu lato*) isolate RC15 showed 100% (ITS), 99.7% (TEF-1α), and 99.5% (RPB2) identity to *Neocosmospora pseudensiformis* (*Fusarium solani* species complex), as shown in Supplementary Table S4.

**Figure 2:**
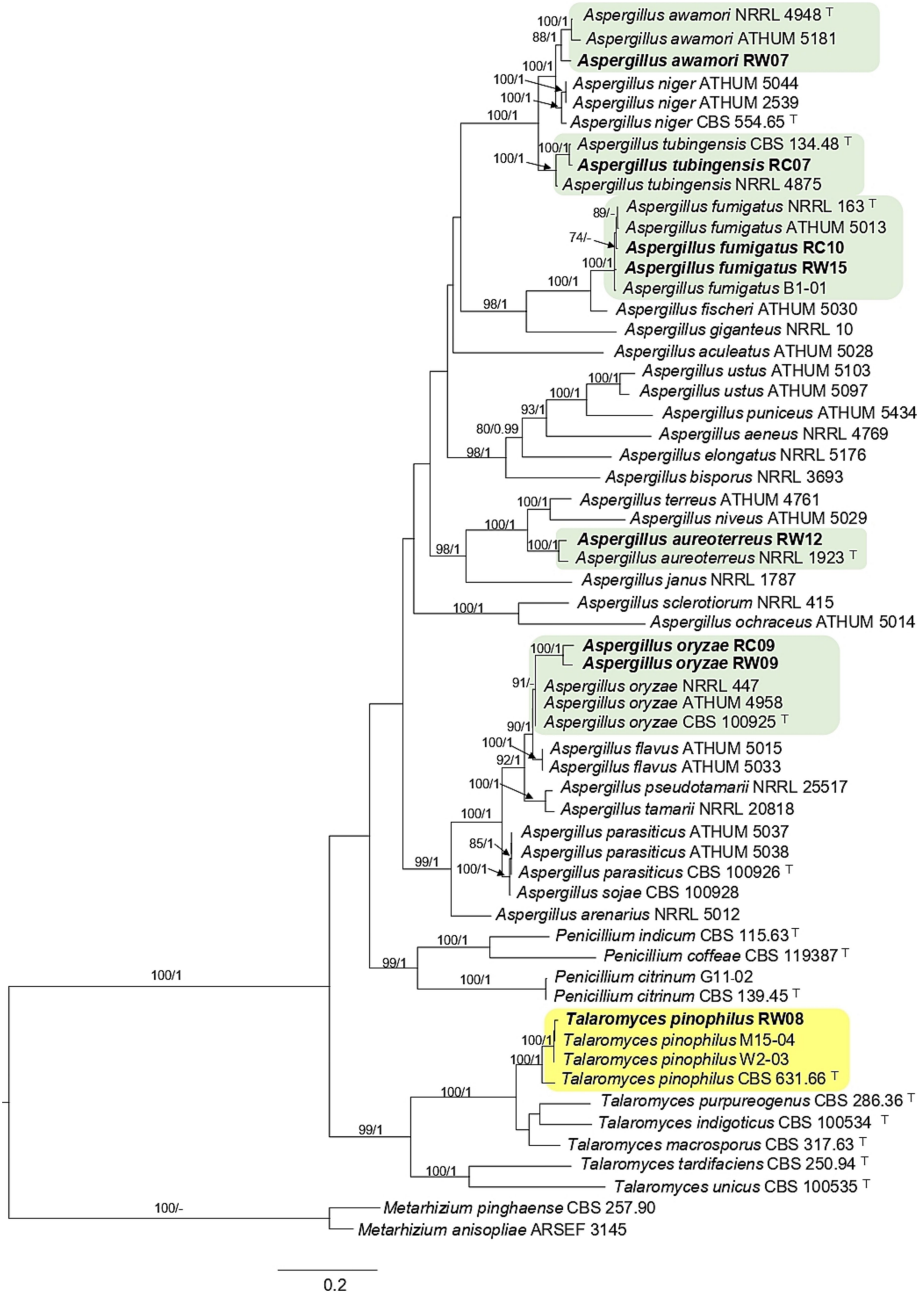
Maximum-likelihood (ML) phylogeny inferred from concatenated ITS, BenA, CaM, and RPB2 loci for eight representative isolates and related reference taxa. Node support values are presented as maximum-likelihood bootstrap percentages (ML, left; ≥70%) and Bayesian posterior probabilities (BPP, right; ≥0.95). Isolates generated in this study are highlighted in bold, with type strains of reference species designated with a superscript “T”. The tree was rooted with *Metarhizium anisopliae* and *M. pinghaense* as outgroup taxa.

**Figure 3:**
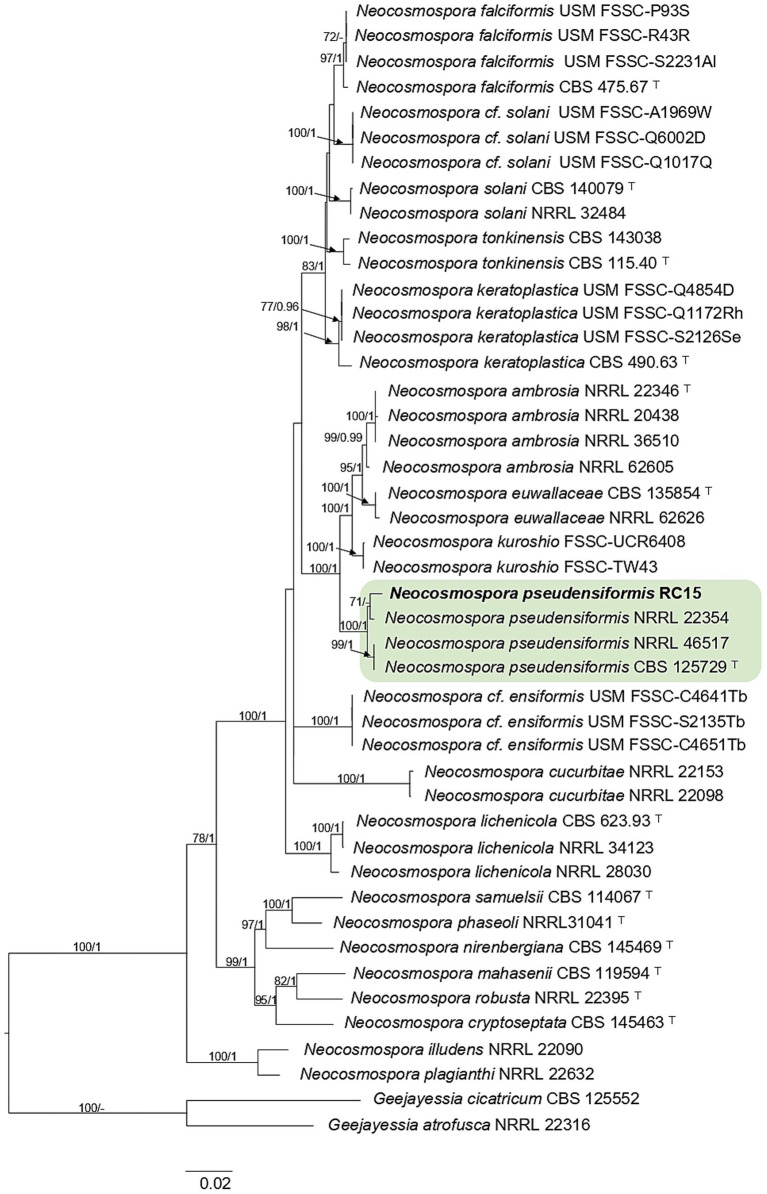
Maximum-likelihood (ML) phylogeny inferred from concatenated ITS, TEF-1α, and RPB2 loci for each fungal isolate and related reference taxa. Node support values are shown as ML bootstrap percentages (BS, left; ≥70%) and Bayesian posterior probabilities (BPP, right; ≥0.95). The isolates generated in this study are highlighted in bold, with type strains indicated by a superscript “T.” The tree was rooted with *Geejayessia cicatricum* and *G. atrofusca* as outgroup taxa.

Phylogenetic inference using maximum likelihood (RAxML) and Bayesian analysis (MrBayes 3.2.7 (44)) recovered topologies with moderate to high support that were consistent with BLAST-based identifications and morphological classification. In the multi-locus phylogeny, RW12 was clustered with the reference strains of *A. aureoterreus* (ML = 100%, PP = 1.00; Figure 2). Two isolates (RC10 and RW15) were grouped with *A. fumigatus* (ML = 100%, PP = 1.00; Figure 2), whereas RC09 and RW09 were clustered with *A. oryzae* (ML = 91%; Figure 2). RW07 was grouped with *A. awamori* (ML = 88%, PP = 1.00; Figure 2), and RC07 was clustered with *A. tubingensis* (ML = 100%, PP = 1.00; Figure 2). Notably initially, isolated RW08 was identified morphologically as *Penicillium*, but was resolved as *Talaromyces* and was clustered strongly with *T. pinophilus* (ML = 100%, PP = 1.00; Figure 2). The *Fusarium* (*sensu lato*) isolate RC15 formed a strongly supported clade with *Neocosmospora pseudensiformis* (ML = 100%, PP = 1.00; Figure 3), confirming its placement.

Overall, the multi-locus sequencing and phylogenetic analyses of the representative isolates indicated that *Aspergillus* was the predominant genus recovered from the rice samples, comprising five species (*A. aureoterreus*, *A. awamori*, *A. oryzae*, *A. fumigatus*, and *A. tubingensis*), alongside *Talaromyces pinophilus* and *Neocosmospora pseudensiformis*.

### 3.4 Mycotoxin occurrence in rice samples

Analysis of 50 rice grain samples (25 unpolished and 25 polished) by LC–MS/MS revealed the presence of beauvericin (BEA; *n* = 2) and zearalenone (ZEN; *n* = 2) exclusively in unpolished rice. ZEN was detected in two unpolished rice samples at concentrations of 39.42 and 65.57 μg/kg, while BEA was detected in two unpolished rice samples at 0.85 and 1.77 μg/kg. Whereas no targeted mycotoxins were detected in polished rice samples above the method detection limits. Overall, mycotoxin occurrence was limited to a small proportion of unpolished rice samples, with no detectable contamination in polished rice.

## 4 Discussion

Morphological characterization is an important first step in identifying fungi isolated from rice grains. Colony appearance, spore coloration, and the structures of conidiophores, vesicles, and conidia are highly informative for genus-level identification, especially for the common food-related genera (*Aspergillus*, *Penicillium*, and *Fusarium*) (14, 32, 52). In the present study, fungal contamination was assessed using the blotter method, revealing a higher incidence of fungi in the unpolished rice (13.28%) than in the polished rice (11.92%). This pattern aligned with prior reports indicating that the outer layers of rice grains serve as major reservoirs for fungal spores. The bran layer, comprising the pericarp, testa, aleurone layer, and germ, contains the highest concentrations of lipids, proteins, and minerals and its loosely disrupted cellular structure created during milling facilitates the adhesion and growth of fungi more effectively than the endosperm (53–55). Postharvest factors, such as grain moisture, delays in drying, and storage conditions, further enhance fungal proliferation, particularly among storage-associated fungi such as *Aspergillus* spp. which grow rapidly at water activity values above 0.70–0.80 (10, 56). In contrast, *Fusarium* species are primarily field-associated fungi, with infection and mycotoxin production occurring typically during the pre-harvest stage under favorable climatic conditions (57).

Often, in tropical regions, *Aspergillus* spp. are predominant and are frequently reported as contaminants of rice, maize, legumes, and rice bran consistent with their ability to grow under warm and humid storage environments. Well-known toxigenic species include *A. flavus* and *A. parasiticus*, which produce Aflatoxins, classified as Group 1 carcinogens by IARC, which are common in improperly stored unpolished rice and rice bran (58). Reddy et al. (59) identified Asia as a hotspot for *A. flavus* and AFs contamination due to climate suitability and co-occurrence with other fungi during harvest and postharvest stages. Additionally, Asghar et al. (60) highlighted moisture control as the most critical factor for inhibiting *Aspergillus* growth and reducing AFs production in rice. However, *A. flavus* and *A. parasiticus* were not detected in the present study, while in addition, AFs were not found in the rice samples analyzed.

The genus *Talaromyces* has gained increasing attention in food safety due to its occurrence in rice, rice bran, and other high-moisture cereals. Several species are known to synthesize emerging mycotoxins. In particular, recently, *Talaromyces pinophilus* has been considered a potential producer of emerging mycotoxin, raising concerns regarding possible health risks, especially in populations with traditionally high rice consumption (47).

By contrast, *Penicillium* species are associated typically with cereal storage, particularly in cool and low-moisture conditions, although they may also occur in rice stored for extended periods or managed under suboptimal moisture levels. The most notable species, *P. citrinum*, produces CIT, a nephrotoxic compound. Fleurat-Lessard et al. (61) emphasized the role of *Penicillium* as a major storage fungus in cereals, and da Rocha et al. (62) linked the occurrence of CIT in various grains to poor moisture control.

In contrast to the other fungal groups identified in this study, *Fusarium* species are associated primarily with the preharvest contamination of cereals. This ecological characteristic is consistent with the present findings, in which *Fusarium* was detected exclusively in unpolished rice. Species such as *F. verticillioides*, *F. proliferatum* and *F. graminearum*, and are known producers of FBs, DON, and ZEN, which originate typically in the field under cool and moist conditions during crop development. Rice cultivated in humid tropical regions has been reported to be particularly susceptible to *Fusarium* infection and toxin contamination (13). In the present study, further supported the notion that *Fusarium*-related contamination was largely associated with the outer grain layers and preharvest conditions, rather than postharvest storage environments (63–65).

Our morphological findings were consistent with widely reported patterns of fungal contamination in rice. *Aspergillus* was the predominant genus, representing ~90% of the isolates recovered, followed by *Talaromyces* and *Fusarium*. This trend aligned with a report from Thailand indicating that *Aspergillus* and *Penicillium/Talaromyces* are among the major fungal contaminants of rice (47). Similar observations have been reported across Asia, where *Fusarium* and *Aspergillus* can occur throughout the rice production chain, from paddy rice to milled products (66, 67). Collectively, these studies indicated that *Aspergillus*, *Penicillium/Talaromyces*, and *Fusarium* represent key fungal groups associated with rice contamination, highlighting the importance of species-level identification, particularly for taxa with mycotoxin-producing potential. However, morphology alone often provides insufficient resolution for species delimitation because of the phenotypic similarity among *Aspergillus*, *Penicillium*, and *Talaromyces*, phenotypic plasticity under different culture conditions, and the frequent occurrence of cryptic species. Consequently, reliable species identification typically requires molecular approaches (68). Therefore, polyphasic taxonomy is now standard practice in fungal systematics and integrating morphology with multi-locus sequence data is used widely to achieve reliable species identification (69). Typically, multi-locus analyses combine markers with different evolutionary rates, including ITS as a universal barcode, and protein-coding genes such as BenA (*β*-tubulin), CaM (calmodulin), and RPB2 (the second-largest subunit of RNA polymerase II), which provide a complementary phylogenetic signal. Often, in *Aspergillus* and *Penicillium*, ITS is informative at the genus level but frequently lacks resolution for species delimitation, whereas BenA and CaM offer higher discriminatory power and RPB2 often yields comparatively stable phylogenetic reconstruction (52, 70). Combining multiple-loci can reduce single-gene incongruence and increase confidence when clades are supported by maximum-likelihood bootstrap values and Bayesian posterior probabilities (32, 70). In the present study, the BLAST similarity searches broadly supported the morphological assignments for most isolates. For example, isolate RW12 showed ITS, BenA, CaM, and RPB_2_ sequence similarities of 100, 99.6, 97.6, and 98.6%, respectively, confirming its identity as *A. aureoterreus*. The isolate RC15, initially assigned to *Fusarium* based on morphology, showed ITS, TEF-1α, and RPB2 sequence similarities of 100, 99.7, and 99.5%, respectively, to *Neocosmospora pseudensiformis*. This species has undergone recent taxonomic revision and is currently placed in the genus *Neocosmospora*, following the reclassification of members of the *Fusarium solani* species complex based on multi-locus phylogenetic analyses (71). Phylogenetic analyses based on multi-locus datasets (ITS, BenA, CaM, RPB_2_ for *Aspergillus/Talaromyces* and ITS, TEF-1α, RPB_2_ for *Neocosmospora*) provided robust species-level resolution, with most nodes receiving moderate to high maximum-likelihood bootstrap and Bayesian support. RW12 was clustered with *A. aureoterreus* (ML = 100%, PP = 1.00), while RW08, morphologically classified as *Penicillium*, was reassigned to *Talaromyces*, specifically *T. pinophilus* (ML = 100%, PP = 1.00). The phylogenetic analyses in this study placed the isolates within the lineage currently recognized as *Neocosmospora* (72). Although the taxonomic status of the *Fusarium solani* species complex (FSSC) remains debated (16), our results are consistent with recent taxonomic frameworks. Overall, these results reinforced the value of a polyphasic framework that integrates morphology with sequence similarity and phylogenetic inference for accurate species identification (69).

Mycotoxin contamination remains a major food safety concern in rice, particularly for *Fusarium*-associated toxins reported worldwide. Although a wide range of *Fusarium* mycotoxins has been described in cereals, the present study focused on a targeted LC–MS/MS analysis of selected regulated and emerging mycotoxins in rice samples. The occurrence of BEA in unpolished rice was consistent with published market surveys, including Laut et al. (47), which reported BEA contamination in commercial rice samples. BEA is recognized increasingly as an emerging mycotoxin frequently associated with *Fusarium* contamination in cereals (73). Other studies have documented the co-occurrence of *Fusarium* toxins, including trichothecenes and ZEN, in rice and rice by-products such as husk, bran, broken rice, and discolored kernels (74), indicating that mycotoxin contamination is strongly influenced by milling practices and postharvest processing. In addition, Monteiro et al. (67) demonstrated that unpolished and paddy rice tend to have substantially higher mycotoxin burdens than polished rice, supporting the observation in the present study that mycotoxins were detected exclusively in unpolished rice. Consistent with this, our study detected BEA and ZEN exclusively in unpolished rice, whereas Nandinidevi et al. (75) study primarily reported OTA, with higher contamination levels in unpolished rice than in polished rice. Although the specific mycotoxins detected differed between the two studies, both consistently indicate that unpolished rice is more susceptible to mycotoxin contamination than polished rice. This pattern underscores that the rice bran fraction may serve as a major reservoir for fungal colonization and mycotoxin accumulation. Although *Fusarium* was isolated only from unpolished rice and molecularly identified as *Neocosmospora pseudensiformis*, no fungal toxin production test was performed in this study. Accordingly, the detection of BEA and ZEN in rice grains most likely reflects field-derived contamination occurring before harvest and/or contributions from the broader *Fusarium* (*sensu lato*) community in the production environment. The preferential accumulation of mycotoxins in the bran fraction further supported the hypothesis that rice bran serves as a primary site for toxin retention, explaining the elevated risk associated with unpolished rice.

In the present study, ZEN was detected at low levels (39.42 and 65.57 μg/kg) in only two unpolished rice samples, whereas no ZEN was found in polished rice above the detection limits. These concentrations were far below the former EU reference limit of 100 μg/kg (EC No 1881/2006) (76). At present, Thailand has no regulatory maximum level for ZEN in rice (77), and the updated EU Regulation 2023/915 does not specify a ZEN limit for rice (78). Although consumer exposure appears negligible in this study, the exclusive detection of ZEN in unpolished rice further supports the role of bran as a hotspot for *Fusarium* toxin retention and underscores the need for continued surveillance. BEA was detected at very low levels (0.85 and 1.77 μg/kg) in only two unpolished rice samples, whereas no BEA was found in polished rice above the detection limits. Currently, no regulatory maximum level has been established for BEA, and surveys of cereals commonly report contamination below 100 μg/kg (79). However, markedly higher concentrations have been reported in certain regions, such as Morocco, where BEA levels in rice ranged from 3.8 to 26.3 mg/kg, with a maximum of 26.3 mg/kg. This contrast highlights strong geographical and agronomic influences on BEA occurrence and supports the need for continued monitoring of this emerging mycotoxin in rice (80).

Overall, *Aspergillus*, *Talaromyces*, and *Fusarium* (*sensu lato*; including taxa currently placed in *Neocosmospora*) were the predominant genera contaminating rice in the present study, consistent with global trends and the findings of Siri-Anusornsak et al. (81), who reported that these genera are important sources of regulated, emerging, and modified mycotoxins in Southeast Asian cereals. A limitation of this study is that not all isolates were subjected to molecular identification. Although representative isolates were selected for multi-locus sequencing, morphology-based identification alone may not provide sufficient resolution for closely related or crytic sprcies, particularly within Aspergillus and *Penicillium/Talaromyces*. Future studies should incorporate molecular identification for all isolates to improve taxonomic resolution. Unpolished rice had higher contamination than polished rice, reflecting the nutrient-rich composition of bran and its role as a hotspot for fungal colonization and toxin synthesis. Previous surveys in Thailand have reported frequent mycotoxin contamination in rice, including AFs, BEA and ZEN (45), However, in the present study, only BEA and ZEN were detected in rice samples. This discrepancy likely reflects differences in sampling frame, geographical origin, seasonality, post-harvest handling, and the targeted analyte panel and detection limits applied. The rice sample analyzed in this study covered a range of brands, rice types, producers, and distributors commonly available in retail department stores. However, the sampling was limited to retail stores in Bangkok and may not fully represent all rice products across the country. In addition, the study period was limited and may not reflect seasonal variation. Further studies with larger sample sizes are needed. Collectively, these observations emphasized the central role of the bran layer in fungal and mycotoxin accumulation and reinforced the findings of the present study. Future research should integrate multi-mycotoxin profiling with post-harvest environmental modeling to clarify how moisture dynamics, degree of milling, and storage micro-conditions drive toxin accumulation, thereby enabling the development of early-warning indicators for mycotoxin risk assessment in rice.

## 5 Conclusion

In conclusion, the results of this study confirmed that unpolished rice may contain a variety of fungi species originating from the environment, as well as handling during harvesting, sorting and packaging. As expected, polished rice due to processing steps had a lower abundance of culturable fungal species. The occurrence of mycotoxin was limited to a small percentage of unpolished rice samples. No regulated carcinogenic mycotoxins such as AFs, OTA, or FB1 were found. Using validated LC-MS/MS methods, only two *Fusarium* toxins, ZEN and BEA, were found at low levels in unpolished rice samples, and no mycotoxins above the LOQ were detected in any polished rice. The result suggest low contamination in the rice samples from Bangkok retail markets. The multi-toxin analytical method described enables routine, validated monitoring of rice from various harvests, helping to support food safety objectives.

## Data Availability

The datasets generated during the current study have been deposited in the NCBI database under accession number: https://www.ncbi.nlm.nih.gov/bioproject/PRJNA1470844/. The accession number(s) are provided in the Supplementary materials.
