# Supplementary material for: Fungal diversity and mycotoxins in retail polished and unpolished rice in Thailand
Source: Front Nutr. 2026 Jun 25;13:1828938. doi: 10.3389/fnut.2026.1828938 (PMC13348275; doi:10.3389/fnut.2026.1828938)
Supplement: Supplementary file 1 [file Table_1.DOCX]

Supplementary Material

**Table S1**. *Aspergillus*, *Penicillium*, *Talaromyces*, and related taxa used in multi-locus phylogenetic analyses with corresponding GenBank accession numbers

| **Taxon** | **Strain number** | **NCBI GenBank accession number** | | | |
| --- | --- | --- | --- | --- | --- |
|  |  | **ITS** | ***BenA*** | ***CaM*** | ***RPB2*** |
| *A. aculeatus* | ATHUM 5028 | EU982028 | EU982087 | N/A | N/A |
| *A. aeneus* | NRRL 4769 | EF652474 | EF652298 | N/A | EF652210 |
| *A. arenarius* | NRRL 5012 | EU021615 | EU021674 | N/A | EU021653 |
| *A. awamori* | ATHUM 5181 | EU982010 | EU982069 | N/A | EU982094 |
| *A. awamori* | NRRL 4948 ^T^ | KF288139 | KF288129 | KF288119 | N/A |
| ***A. awamori*** | **RW07** | **PX494414** | **PX852715** | **PX852721** | **PX852730** |
| *A. aureoterreus* | NRRL 1923 ^T^ | EF669580 | EF669524 | EF669538 | EF669622 |
| ***A. aureoterreus*** | **RW12** | **PX494411** | **PX852712** | **PX852718** | **PX852727** |
| *A. bisporus* | NRRL 3693 | EF661208 | EF661121 | N/A | EF661077 |
| *A. elongatus* | NRRL 5176 | EF652502 | EF652326 | N/A | EF652238 |
| *A. fischeri* | ATHUM 5030 | EU982016 | EU982075 | N/A | EU982100 |
| *A. flavus* | ATHUM 5015 | EU982011 | EU982070 | N/A | EU982095 |
| *A. flavus* | ATHUM 5033 | EU982012 | EU982071 | N/A | EU982096 |
| *A. fumigatus* | ATHUM 5013 | EU982013 | EU982072 | N/A | EU982097 |
| *A. fumigatus* | B1-01 | OP946393 | OP965325 | OP980970 | OP965309 |
| *A. fumigatus* | NRRL 163 ^T^ | EF669931 | EF669791 | EF669860 | EF669719 |
| ***A. fumigatus*** | **RC10** | **PX494416** | **N/A** | **PX852723** | **PX852731** |
| ***A. fumigatus*** | **RW15** | **PX494417** | **N/A** | **PX852724** | **PX852732** |
| *A. giganteus* | NRRL 10 | EF669928 | EF669789 | N/A | EF669716 |
| *A. janus* | NRRL 1787 | EU021598 | EU014076 | N/A | EF669620 |
| *A. niger* | ATHUM 2539 | EU982009 | EU982068 | N/A | EU982092 |
| *A. niger* | ATHUM 5044 | EU982008 | EU982067 | N/A | EU982093 |
| *A. niger* | CBS 554.65 ^T^ | AJ223852 | AY585536 | AJ964872 | N/A |
| *A. niveus* | ATHUM 5029 | EU982023 | EU982082 | N/A | EU982105 |
| *A. ochraceus* | ATHUM 5014 | EU982029 | EU982088 | N/A | EU982111 |
| *A. oryzae* | ATHUM 4958 | EU982022 | EU982081 | N/A | EU982104 |
| *A. oryzae* | CBS 100925 ^T^ | MF668185 | EF203138 | EF202055 | N/A |
| *A. oryzae* | NRRL 447 | EF661560 | EF661483 | EF661506 | EF661438 |
| ***A. oryzae*** | **RC09** | **PX494412** | **PX852713** | **PX852719** | **PX852728** |
| ***A. oryzae*** | **RW09** | **PX494413** | **PX852714** | **PX852720** | **PX852729** |
| *A. parasiticus* | ATHUM 5037 | EU982020 | EU982079 | N/A | N/A |
| **Taxon** | **Strain number** | **NCBI GenBank accession number** | | | |
|  |  | **ITS** | ***BenA*** | ***CaM*** | ***RPB2*** |
| *A. parasiticus* | ATHUM 5038 | EU982021 | EU982080 | N/A | N/A |
| *A. parasiticus* | CBS 100926 ^T^ | KY937933 | EF203155 | EF202043 | N/A |
| *A. pseudotamarii* | NRRL 25517 | AF272574 | EF203125 | EF202030 | EU021631 |
| *A. puniceus* | ATHUM 5434 | EU982019 | EU982078 | N/A | EU982101 |
| *A. sclerotiorum* | NRRL 415 | EF661400 | EF661337 | N/A | EF661287 |
| *A. sojae* | CBS 100928 | MF668186 | EF203168 | EF202041 | N/A |
| *A. tamarii* | NRRL 20818 | AF004929 | EF661474 | EF661526 | EU021629 |
| *A. terreus* | ATHUM 4761 | EU982024 | EU982083 | N/A | EU982106 |
| *A. tubingensis* | CBS 134.48 ^T^ | AJ223853 | AY820007 | AJ964876 | N/A |
| *A. tubingensis* | NRRL 4875 | EF661193 | EF661086 | EF661151 | EF661055 |
| ***A. tubigensis*** | **RC07** | **PX494415** | **PX852716** | **PX852722** | **N/A** |
| *A. ustus* | ATHUM 5097 | EU982026 | EU982085 | N/A | EU982108 |
| *A. ustus* | ATHUM 5103 | EU982027 | EU982086 | N/A | EU982109 |
| *Metarhizium anisopliae* | ARSEF 3145 | AF218207 | AY995134 | N/A | DQ522453 |
| *M. pinghaense* | CBS 257.90 | MH862210 | EU248820 | N/A | EU248930 |
| *P. citrinum* | CBS 139.45 ^T^ | MH856132 | GU944545 | N/A | JF417416 |
| *P. citrinum* | G11-02 | OP946395 | OP965326 | N/A | OP965318 |
| *P. coffeae* | CBS 119387 ^T^ | AY742702 | KJ834443 | AY741747 | JN121436 |
| *P. indicum* | CBS 115.63 ^T^ | AY742699 | EU427263 | AY741744 | JN406640 |
| *T. indigoticus* | CBS 100534 ^T^ | NR137076 | JX494308 | KF741931 | N/A |
| *T. macrosporus* | CBS 317.63 ^T^ | NR145155 | JX091382 | KF741952 | KM023292 |
| *T. pinophilus* | CBS 631.66 ^T^ | JN899382 | JX091381 | KF741964 | KM023291 |
| *T. pinophilus* | M15-04 | OP946401 | OP965329 | OP980976 | OP965315 |
| *T. pinophilus* | W2-03 | OP946402 | OP965330 | OP980977 | OP965319 |
| ***T. pinophilus*** | **RW08** | **PX494410** | **PX852711** | **PX852717** | **PX852726** |
| *T. purpureogenum* | CBS 286.36 ^T^ | JN899372 | N/A | JX315655 | JX315709 |
| *T. tardifaciens* | CBS 250.94 ^T^ | KF984874 | KF984560 | KF984682 | KF984908 |
| *T. unicus* | CBS 100535 ^T^ | NR_157429 | KJ865735 | KJ885283 | MN969150 |

Newly generated sequences and tentatively assigned species names based on gross morphological identification in this study are indicated in bold. N/A denotes data not available, and T indicates ex-type strains. ATHUM refers to the Culture Collection of Fungi, National and Kapodistrian University of Athens; NRRL refers to the National Center for Agricultural Utilization Research; and CBS refers to the culture collection of the CBS-KNAW Fungal Biodiversity Centre, Utrecht, the Netherlands.

**Table S2**. *Neocosmospora* and related taxa used in multi-locus phylogenetic analyses with corresponding GenBank accession numbers.

| **Taxon** | **strain number** | **NCBI GenBank accession number** | | | |
| --- | --- | --- | --- | --- | --- |
|  |  | **ITS** | | **EF-1** | **RPB2** |
| *Geejayessia atrofusca* | NRRL 22316 | AF178423 | AF17831 | | JX171609 |
| *G. cicatricum* | CBS 125552 | MH863560 | HM626644 | | HQ728153 |
| *N. ambrosia* | NRRL 20438 | AF178397 | AF178332 | | JX171584 |
| *N. ambrosia* | NRRL 22346 ^T^ | EU329669 | FJ240350 | | EU329503 |
| *N. ambrosia* | NRRL 36510 | KC691558 | KC691530 | | KC691648 |
| *N. ambrosia* | NRRL 62605 | KC691559 | KC691531 | | KC691649 |
| *N. cf. solani* | USM FSSC-A1969W | KC009603 | KC161397 | | N/A |
| *N. cf. solani* | USM FSSC-Q1017Q | KC009605 | KC161399 | | N/A |
| *N. cf. solani* | USM FSSC-Q6002D | KC009620 | KC161414 | | N/A |
| *N. cryptoseptata* | CBS 145463 ^T^ | NR 172368 | AF178351 | | EU329510.1 |
| *N. cucurbitae* | NRRL 22098 | DQ094301 | AF178327 | | EU329489 |
| *N. cucurbitae* | NRRL 22153 | DQ094302 | AF178346 | | EU329492 |
| *N. euwallaceae* | CBS 135854 ^T^ | JQ038014 | JQ038007 | | JQ038028 |
| *N. euwallaceae* | NRRL 62626 | KC691560 | KC691532 | | KU171702 |
| *N. cf. ensiformis* | USM FSSC-C4641Tb | KC009590 | KC161384 | | N/A |
| *N. cf. ensiformis* | USM FSSC-C4651Tb | KC009589 | KC161383 | | N/A |
| *N. cf. ensiformis* | USM FSSC-S2135Tb | KC009588 | KC161382 | | N/A |
| *N. falciformis* | CBS 475.67 ^T^ | MG189935.1 | LT906669.1 | | LT960558 |
| *N. falciformis* | USM FSSC-P93S | KF836674 | KF836702 | | N/A |
| *N. falciformis* | USM FSSC-R43R | KF836673 | KF836701 | | N/A |
| *N. falciformis* | USM FSSC-S2231Al | KF836672 | KF836700 | | N/A |
| *N. nirenbergiana* | CBS 145469 ^T^ | AF178403 | AF178339 | | EU329505.1 |
| *N. illudens* | NRRL 22090 | AF178393 | AF178326 | | JX171601.1 |
| *N. keratoplastica* | CBS 490.63 ^T^ | LR583721.1 | LT906670.1 | | LT960562 |
| *N. keratoplastica* | USM FSSC-Q1172Rh | KF836670 | KF836698 | | N/A |
| *N. keratoplastica* | USM FSSC-Q4854D | KF836668 | KF836696 | | N/A |
| *N. keratoplastica* | USM FSSC-S2126Se | KF836669 | KF836697 | | N/A |
| *N. kuroshio* | FSSC-TW43 | MK432868.1 | MK435445.1 | | MK435529 |
| *N. kuroshio* | FSSC-UCR6408 | MK432885.1 | MK435462.1 | | MK435546 |
| *N. lichenicola* | CBS 623.93 ^T^ | LR583730.1 | LR583620.1 | | LR583845.1 |
| *N. lichenicola* | NRRL 28030 | DQ094355 | DQ246877 | | EF470146 |
| *N. lichenicola* | NRRL 34123 | DQ094645 | DQ247192 | | EU329635 |
| *N. mahasenii* | CBS 119594 ^T^ | JF433045 | DQ247513 | | LT960563 |
| **Taxon** | **strain number** | **NCBI GenBank accession number** | | | |
|  |  | **ITS** | **EF-1** | | **RPB2** |
| *N. phaseoli* | NRRL 31041 ^T^ | AY220239 | AY220193 | | JX171643.1 |
| *N. plagianthi* | NRRL 22632 | AF178417 | AF178354 | | JX171614.1 |
| *N. pseudensiformis* | NRRL 22354 | AF178402 | AF178338 | | EU329504 |
| *N. pseudensiformis* | NRRL 46517 | KC691584.1 | KC691555.1 | | KC691674 |
| *N. pseudensiformis* | CBS 125729 ^T^ | JF433037 | DQ247512 | | KC691645 |
| ***N. pseudensiformis*** | **RC15** | **PX494409** | **PX738485** | | **PX852725** |
| *N. samuelsii* | CBS 114067 ^T^ | LR583764 | LR583644 | | LR583874.1 |
| *N. solani* | NRRL 32484 | DQ094449 | DQ246982 | | EU329583 |
| *N. solani* | CBS 140079 ^T^ | KT313633 | KT313611 | | KT313623 |
| *N. tonkinensis* | CBS 115.40 ^T^ | MG189941 | LT906672 | | LT960564 |
| *N. tonkinensis* | CBS 143038 | MG189942 | LT906673 | | LT960565 |
| *N. robusin* | NRRL 22395 ^T^ | NR_172367 | AF178341 | | EU329507.1 |

Newly generated sequences and tentatively assigned species names based on gross morphological identification in this study are indicated in bold. N/A denotes data not available, and T indicates ex-type strains. ATHUM refers to the Culture Collection of Fungi, National and Kapodistrian University of Athens; NRRL refers to the National Center for Agricultural Utilization Research; CBS refers to the culture collection of the CBS-KNAW Fungal Biodiversity Centre, Utrecht, the Netherlands; and USM refers to the Culture Collection of Universiti Sains Malaysia.

**Table S3.** BLAST search and phylogenetic analyses based on target genes for *Aspergillus* and *Penicillium/Talaromyces* spp. isolates

| **Original Code** | **Morphological Identification** | **The closest taxon based on BLAST similarity** | | | | | | | | **Proposed fungal taxon (ID) based on sequene based data and phylogenetic tree** |
| --- | --- | --- | --- | --- | --- | --- | --- | --- | --- | --- |
|  |  | **ITS** | | ***RPB2*** | | ***BanA*** | | ***CaM*** | |  |
|  |  | **Putative taxonomic affinity** | **Identity**  **(%)** | **Putative taxonomic affinity** | **Identity**  **(%)** | **Putative taxonomic affinity** | **Identity**  **(%)** | **Putative taxonomic affinity** | **Identity**  **(%)** |  |
| RC07 | *Aspergillus* sp. | *Aspergillus* sp. | 99.8 | ND | ND | *A. tubingensis* | 99.6 | *A. tubingensis* | 99.7 | *A. tubingensis* |
| RC09 | *Aspergillus* sp. | *A. flavus* | 100 | *A. oryzae* | 99.9 | *A. flavus* | 99.6 | *A. flavus* | 99.9 | *A. oryzae* |
| RC10 | *A. fumigatus* | *A. fumigatus* | 99.5 | *A. fumigatus* | 100 | ND | ND | *A. fumigatus* | 99.8 | *A. fumigatus* |
| RW07 | *Aspergillus* sp. | *A. niger* | 99.7 | *A. niger* | 100 | *A. awamori* | 99.9 | *A. awamori* | 99.9 | *A. awamori* |
| RW08 | *Penicillium sp./ Talaromyces sp.* | *T. pinophilus* | 99.8 | *Talaromyces* sp*.* | 99.9 | *T. pinophilus* | 100 | *T. pinophilus* | 99.4 | *T. pinophilus* |
| RW09 | *Aspergillus* sp. | *A. flavus* | 99.8 | *A. oryzae* | 99.9 | *A. flavus* | 98.9 | *A. oryzae* | 99.7 | *A. oryzae* |
| RW12 | *Aspergillus* sp. | *A. aureoterreus* | 100 | *A. aureoterreus* | 98.6 | *A. aureoterreus* | 99.6 | *A. aureoterreus* | 97.6 | *A. aureoterreus* |
| RW15 | *A. fumigatus* | *A. fumigatus* | 99.7 | *A. fumigatus* | 100 | ND | ND | *A. fumigatus* | 99.8 | *A. fumigatus* |

ND indicates not detected.

**Table S4**. BLAST search and phylogenetic analyses based on target genes for *Fusarium* isolates.

| **Original Code** | **Morphological Identification** | **The closest taxon based on BLAST similarity** | | | | | | **Proposed fungal taxon (ID) based on sequene based data and phylogenetic tree** |
| --- | --- | --- | --- | --- | --- | --- | --- | --- |
|  |  | **ITS** | | ***TEF-1α*** | | ***RPB2*** | |  |
|  |  | **Putative taxonomic affinity** | **Identity**  **(%)** | **Putative taxonomic affinity** | **Identity**  **(%)** | **Putative taxonomic affinity** | **Identity**  **(%)** |  |
| RC15 | *Fusarium* sp. | *Fusarium* sp*.* | 100 | *F. pseudensiforme* | 99.7 | *F. pseudensiforme* | 99.5 | *F. pseudensiforme* |

Note: Species identification was performed using BLAST searches of ITS, TEF-1α, and RPB2 sequences and confirmed by multi-locus phylogenetic analyses. Taxonomic names follow current nomenclature, in which some species formerly classified as *Fusarium* are now placed in *Neocosmospora*.

**Table S5.** Optimized MS/MS parameters for the determination of 15 mycotoxins by LC–MS/MS

| **Analyte** | **Precusor ion (m/z)** | **Product ions (m/z)** | **Collision energy (eV)** | **Fragmentor (V)** | **Retention time (min)** | **Polarity** |
| --- | --- | --- | --- | --- | --- | --- |
| AFB_1_ | 313.07 | 285.1  241.0^a^ | 21  35 | 150  150 | 6.32 | Positive |
| AFB_2_ | 315.09 | 287.1  259.0^a^ | 25  29 | 160  160 | 6.18 | Positive |
| AFG_1_ | 329.07 | 311.0  243.0^a^ | 25  43 | 160  160 | 6.16 | Positive |
| AFG_2_ | 331.08 | 313.0  245.0^a^ | 25  29 | 180  180 | 6.02 | Positive |
| OTA | 404.00 | 192.9  102.1^a^ | 48  80 | 130  130 | 6.79 | Positive |
| T_2_ | 489.40 | 387.3  245.2^a^ | 20  26 | 170  170 | 7.00 | Positive |
| DAS | 384.20 | 307.1  199.0^a^ | 5  13 | 60  60 | 6.28 | Positive |
| ZEA | 319.16 | 283.0  187.0^a^ | 5  17 | 80  80 | 7.24 | Positive |
| FB_1_ | 722.40 | 352.5  334.4^a^ | 40  45 | 160  160 | 5.59 | Positive |
| FB_2_ | 706.30 | 336.2  318.3^a^ | 35  40 | 200  200 | 5.99 | Positive |
| BEA | 801.40 | 784.3  244.1^a^ | 13  35 | 160  160 | 9.13 | Positive |
| CIT | 251.10 | 233.1  205.1^a^ | 3  20 | 120  120 | 5.79 | Positive |
| ALT | 259.10 | 187.9  160.1^a^ | 25  33 | 240  240 | 6.08 | Positive |
| DON | 355.10 | 265.1  59.1^a^ | 4  10 | 90  90 | 3.71 | Negative |
| NIV | 371.10 | 281.0  59.1^a^ | 4  10 | 80  80 | 2.26 | Negative |

^a^ indicates qualitative product ions used for confirmation.

**Table S6**. Accuracy and precision of the proposed LC–MS/MS method for the determination of mycotoxins in polished rice and unpolished rice.

| **Mycotoxins** | **Groups of polished rice** | | | | | | | | | **Groups of unpolished rice** | | | | | | | | |
| --- | --- | --- | --- | --- | --- | --- | --- | --- | --- | --- | --- | --- | --- | --- | --- | --- | --- | --- |
|  | **Recovery (%)**  **(n = 7)** | | | **Intra-day precision (%RSD) (n=7)** | | | **Inter-day precision(%RSD) (n = 21)** | | | **Recovery (%)**  **(n = 7)** | | | **Intra-day precision (%RSD) (n=7)** | | | **Inter-day**  **precision(%RSD) (n = 21)** | | |
|  |  |  |  |  |  |  |  |  |  |  |  |  |  |  |  |  |  |  |
|  |  |  |  |  |  |  |  |  |  |  |  |  |  |  |  |  |  |  |
|  | L1 | L2 | L3 | L1 | L2 | L3 | L1 | L2 | L3 | L1 | L2 | L3 | L1 | L2 | L3 | L1 | L2 | L3 |
| AFB_1_ | 87.8 | 90.4 | 95.6 | 3.8 | 3.3 | 3.4 | 4.2 | 3.9 | 2.1 | 83.3 | 86.8 | 89.5 | 5.5 | 5.7 | 4.4 | 8.1 | 6.7 | 5.9 |
| AFB_2_ | 89.4 | 90.1 | 102.4 | 7.6 | 2.7 | 2.8 | 5.7 | 2.1 | 1.3 | 86.4 | 88.1 | 88.6 | 4.9 | 4.5 | 4.6 | 8.5 | 7.6 | 5.4 |
| AFG_1_ | 90.6 | 92.5 | 98.6 | 7.7 | 5.7 | 2.3 | 6.1 | 2.7 | 2.5 | 85.2 | 87.5 | 93.4 | 5.2 | 4.4 | 5 | 6.2 | 9.1 | 5.8 |
| AFG_2_ | 86.8 | 85.7 | 98.4 | 6.5 | 2.7 | 2.4 | 3.5 | 1.8 | 3.9 | 88.1 | 88.4 | 90.3 | 6.1 | 5.9 | 4.7 | 5.1 | 7.2 | 7.1 |
| OTA | 92.7 | 94.1 | 101.5 | 3.2 | 5.4 | 3 | 3.6 | 2.4 | 5.8 | 92.8 | 90.4 | 91.7 | 3.8 | 4.3 | 2.9 | 8.1 | 7.2 | 4.2 |
| T_2_ | 88.4 | 91.4 | 95.6 | 5.6 | 2.2 | 2.4 | 5.7 | 3 | 6.4 | 81.3 | 84.8 | 88.6 | 4.4 | 3.8 | 3.9 | 7.9 | 7.3 | 3.2 |
| DAS | 89.3 | 94.5 | 97.4 | 4.3 | 2.2 | 2.7 | 4.5 | 2.5 | 5.4 | 85.7 | 87.9 | 90.4 | 3.9 | 4.2 | 4.5 | 5.1 | 5.5 | 4.5 |
| ZEA | 85.6 | 89.2 | 88.6 | 5.2 | 2.2 | 2.5 | 4.2 | 3.4 | 6.4 | 83.4 | 88.3 | 87.9 | 6.7 | 6.4 | 7.5 | 9.1 | 8.4 | 7.5 |
| FB_1_ | 90.3 | 93.3 | 93.5 | 7.4 | 1.6 | 4.4 | 5.5 | 2.3 | 2.2 | 79.5 | 83.7 | 85.6 | 4.5 | 4.9 | 5.1 | 4.8 | 8.1 | 5.9 |
| FB_2_ | 89.5 | 91.7 | 92.2 | 7.7 | 1.8 | 2.6 | 6.2 | 2.4 | 1.7 | 81.4 | 86.6 | 80.2 | 5.3 | 5.1 | 4.8 | 5.4 | 8.6 | 6.6 |
| BEA | 95.5 | 95.7 | 99.2 | 4.9 | 1.7 | 1.8 | 4.6 | 2.8 | 3.2 | 89.7 | 91.8 | 93.4 | 5.3 | 4.1 | 3.6 | 9.3 | 4.9 | 5 |
| CIT | 88.5 | 88.7 | 90.3 | 3.2 | 1.8 | 3.5 | 4.7 | 2.4 | 1.4 | 85.3 | 88.8 | 90.6 | 3.2 | 4.7 | 2.8 | 3.4 | 5.5 | 6.5 |
| ALT | 87.3 | 92.5 | 90.7 | 2.8 | 3.7 | 3 | 4.2 | 2.6 | 2.6 | 86.8 | 89.4 | 92.4 | 4.8 | 4.6 | 3.9 | 4.3 | 5.9 | 5.7 |
| DON | 82.7 | 85.6 | 87.7 | 4.5 | 2.6 | 6.3 | 8.3 | 5.6 | 4.9 | 78.2 | 82.6 | 80.3 | 6.5 | 7.8 | 7.9 | 5.1 | 7.2 | 8.1 |
| NIV | 77.9 | 83.2 | 83.6 | 6.6 | 2.5 | 7.7 | 8.5 | 4.4 | 4.7 | 74.5 | 79.7 | 83.6 | 7.1 | 6.9 | 8.3 | 2.5 | 7 | 5.3 |

Note: L1 (spiking level 1): AFB₁, AFB₂, AFG₁, AFG₂, OTA, and BEA at 1 μg/kg; FB₁, FB₂, and ALT at 2 μg/kg; T2 and CIT at 5 μg/kg; DAS at 10 μg/kg; ZEA and DON at 50 μg/kg; and NIV at 100 μg/kg.

L2 (spiking level 2): AFB₁, AFB₂, AFG₁, AFG₂, OTA, and BEA at 10 μg/kg; FB₁, FB₂, and ALT at 20 μg/kg; T2 and CIT at 50 μg/kg; DAS at 100 μg/kg; ZEA and DON at 250 μg/kg; and NIV at 500 μg/kg.

L3 (spiking level 3): AFB₁, AFB₂, AFG₁, AFG₂, OTA, and BEA at 40 μg/kg; FB₁, FB₂, and ALT at 100 μg/kg; T2 and CIT at 200 μg/kg; DAS at 250 μg/kg; ZEA and DON at 500 μg/kg; and NIV at 1,000 μg/kg
